# A Switched Algorithm for Adaptive Feedback Cancellation Using Pre-Filters in Hearing Aids

**DOI:** 10.3390/audiolres11030037

**Published:** 2021-08-09

**Authors:** Linh Thi Thuc Tran, Sven Erik Nordholm

**Affiliations:** 1 Department of Electrical and Electronics Engineering, Posts and Telecommunications Institute of Technology, Hanoi 12110, Vietnam; 2 Faculty of Science and Engineering, Curtin University, Perth 6102, Australia; S.Nordholm@curtin.edu.au

**Keywords:** Index Terms—adaptive feedback cancellation, prediction error method, NLMS, APA, soft-clipping based stability detector

## Abstract

Acoustic coupling between microphone and loudspeaker is a significant problem in open-fit digital hearing aids. An open-fit compared to a close-fit hearing aid significantly lowers the signal quality and limits the achievable maximum stable gain. Adaptive feedback cancellation (AFC) enables an efficient approach to reduce the impact of acoustic coupling. However, without careful consideration, it can also introduce bias in estimating the feedback path due to the high correlation between the loudspeaker signal and the incoming signal, especially when the incoming signal is spectrally coloured, e.g., speech and music. The prediction error method (PEM) is well known for reducing this bias. The presented study aims to propose a switched PEM with soft-clipping (swPEMSC) that allows for further improvement in convergence/tracking rates, resulting in a better ability to recover from unstable/howling status. This swPEMSC employs a new update rule inspired by a soft-clipping based stability detector (SCSD). It allows to pick up either the PEMSC-NLMS or PEMSC-APA depending on the magnitude of the effective feedback signal; howling corresponds to a large feedback signal. The PEMSC-NLMS with a small step-size ensures a low steady-state error, but slow convergence/tracking rates, while PEMSC-APA with a large step-size allows for fast convergence/tracking rates, but a high steady-state error. By combining those approaches, the proposed approach can take advantage of good characteristics from both. Experimental results using different types of incoming signals and an abrupt change of feedback paths show that the swPEMSC can shorten unstable periods (howling) by improving the convergence and tracking rates while retaining a low steady-state error and good signal quality.

## 1. Introduction

**Figure 1:**
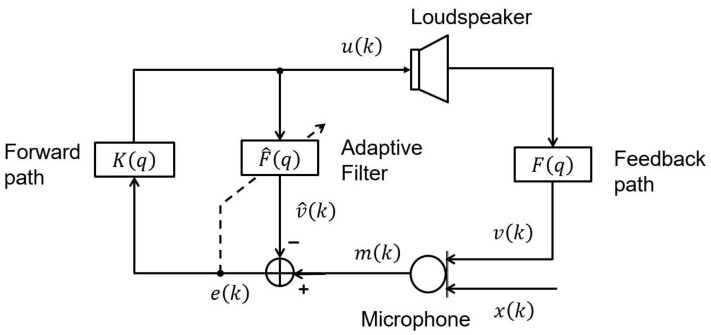
Typical structure of a hearing aid with AFC.

Acoustic feedback occurs due to the acoustic coupling of the loudspeaker signal into the microphone(s). It is a significant problem in public address (PA) systems and open-fit hearing aids (HAs). With the presence of the forward path, the feedback signal is amplified before looping back into the loudspeaker forming a closed-loop system. This feedback signal not only significantly degrades signal quality, but also limits the achievable amplification of those systems. In some particular conditions, it drives the system into an unstable status and howling may occur. The acoustic feedback becomes a more challenging problem for hearing aid applications due to the high demand for small open-fit hearing aids. Many acoustic feedback cancellation methods have been introduced over the last sixty years [1,2,3]. Among them, adaptive feedback cancellation (AFC) has the prominence to reduce the adverse effect of acoustic feedback. This method estimates the acoustic feedback path by using an FIR filter, enabling an estimate of the feedback signal which now can be cancelled from the microphone signal (cf. Figure 1). Due to the closed-loop nature of the HA, the feedback path estimation can produce bias caused by the high correlation between loudspeaker signal and incoming signal [1,2,4].

To reduce this bias, multiple decorrelation methods have been investigated in literature, e.g., delay insertion [1,5], probe noise insertion [6,7,8,9], frequency shifting [10,11], phase modulation [12], and pre-whitening filters [13,14]. Among those methods, prediction error method based adaptive feedback cancellation (PEM-AFC) is well established as it can be effectively applied in both the time domain [14,15,16,17,18,19] and the frequency domain [2,20,21,22,23,24]. In this method, the input signals of an adaptive filter were pre-whitened by using pre-filters, resulting in lower correlation and so bias. Other methods employing sub-band techniques [25,26,27,28], multiple microphones [19,29,30,31,32,33,34], fast-converging adaptive filtering algorithms [14,15,17,32,35,36,37], affine combination of filters [23], and variable step-size (VSS) [11,38,39,40] or combinations of those techniques [31,41,42] for AFC also yielded performance improvement. In [43], an AFC approach based on decomposing a long adaptive filter into a Kronecker Product of two shorter filters has been proposed. Although the above AFC approaches can improve the system performance to a certain degree, the demand for a reliable AFC approach still increases.

In order to further improve the convergence and tracking rates of the previous AFC methods, a hybrid AFC using normalised least mean square algorithm (H-NLMS) has been introduced recently in [44]. The main idea of the H-NLMS was to develop a soft-clipping based stability detector (SCSD) to control the update of the adaptive filter such that the PEMSC-NLMS was chosen during stable periods, otherwise the NLMS algorithm without pre-filters was chosen. This leverages the fact that the NLMS algorithm allows the system to quickly recover from unstable conditions, while the PEMSC-NLMS may provide a lower bias in the estimate of the feedback path.

The purpose of this study is to further improve convergence/tracking rates of the state-of-the-art H-NLMS such that the system can faster recover from unstable/howling status. Inspired by the SCSD, we propose a switched PEM with soft-clipping for AFC in HAs. In contrast to the H-NLMS, first we employ pre-filters to both AFC methods using either the NLMS or the affine projection algorithm (APA) in order to reduce the biased estimate. Then, the SCSD is applied to produce a new update rule for the proposed swPEMSC in such a way that either the PEMSC-NLMS or PEMSC-APA is selected depending on the system converged or unstable, respectively. This is based on an observation that the PEMSC-NLMS with a small step-size provides a low steady-state error, but slow convergence/tracking rates, while the PEMSC-APA with a large step-size obtains much faster convergence/tracking rates, but a high steady-state error. Moreover, PEMSC-APA also faces the trade-off between a low steady-state error and fast convergence/tracking rates when its projection order increases.

Experimental results demonstrate that the proposed method can address those trade-off problems. Specifically, the proposed swPEMSC not only further improves the convergence/tracking rates of the state-of-the-art H-NLMS approach but also achieves a lower steady-state error. It also outperforms other AFC baselines such as PEMSC-NLMS and PEMSC-APA. Furthermore, the speech quality of the proposed swPEMSC is comparable to that of the H-NLMS and much better than the PEMSC-NLMS and PEMSC-APA. Both swPEMSC and H-NLMS obtain very short howling periods occurring at initialisation and at the sudden change of feedback path compared to the PEMSC-NLMS and PEMSC-APA.

Throughout this paper, $E\left\{.\right\}$ and superscript *T* denote expectation and transposition operations, respectively. We use lower and upper letters in bold to represent vectors and matrices, respectively.

The paper is organised as follows. Section 2 describes the structure of a hearing aid and its acoustic feedback problem. Section 3 and Section 4 review the standard AFC method and the PEM-AFC, respectively. The proposed AFC method is presented in Section 5, while the computational complexity of all mentioned methods is compared in Section 6. Experimental results are evaluated in Section 7. Section 8 presents a discussion. Finally, Section 9 concludes the paper.

## 2. Hearing Aids and Acoustic Feedback Problem

A hearing aid is an electronic device that allows for assisting hearing impaired people to restore their hearing capability. A simple HA consists of a loudspeaker (receiver), a microphone, an amplifier, and a battery. The microphone of a HA picks up sound waves from the ambient environment. These picked up sound waves are converted into electrical signals (so-called incoming signals) which are then delayed and amplified through a forward path ($K\left(q\right)$) before reaching HA’s loudspeaker. Finally, the loudspeaker converts the received electrical signals back into sound waves which are fed to the human ear canal. All electrical elements of a HA are powered by a small battery. Due to a presence of a coupling signal (called feedback signal) from loudspeaker to microphone HA suffers from acoustic feedback problem.

**Figure 2:**
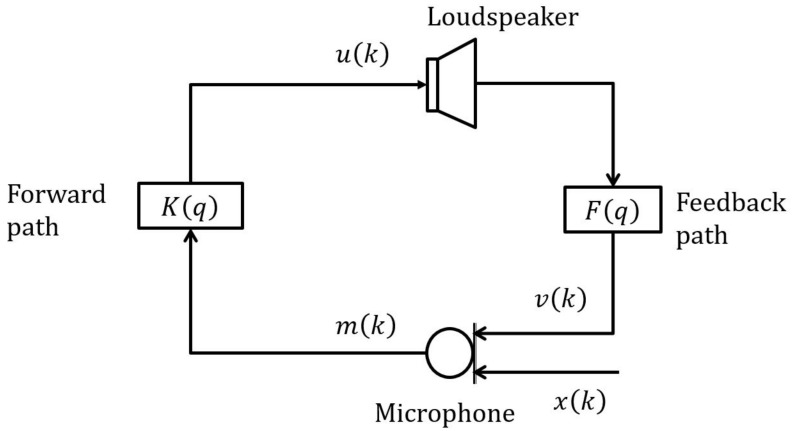
Typical structure of a hearing aid.

Figure 2 illustrates the feedback problem in a HA. The microphone picks up not only the incoming signal but also the acoustic feedback signal, i.e.,

(1) $$m\left(k\right)\;=\;x\left(k\right)\;+\;v\left(k\right),$$

where $m\left(k\right),$ $x\left(k\right)$ and $v\left(k\right)\;=\;F\left(q\right)u\left(k\right)\;=\;f^{T}u\left(k\right)$ denote microphone, incoming and feedback signals, respectively, with $F\left(q\right)$ the polynomial transfer function in *q* of the true feedback path. $F\left(q\right)\;=\;f^{T}q,$ where $f={\left[f_{0},f_{1},\ldots ,f_{L_{f}-1}\right]}^{T}$ is a $L_{f}$-dimensional vector denoting impulse response (IR) of the true feedback path and $q\;=\;{\left[\begin{array}{cccc} 1 & q^{-1} & \ldots & q^{-L_{f}+1} \end{array}\right]}^{T}$ with $q^{-1}$ the discrete-time delay operator. The microphone signal then loops back to the loudspeaker after being processed by a forward path (so-called signal processing path) making HA a closed-loop system. The loudspeaker signal can be expressed as

(2) $$u\left(k\right)\;=\;K\left(q\right)m\left(k\right).$$

In this paper, we assume that $K\left(q\right)\;=\;\left|K\right|q^{-d_{k}}$, where $\left|K\right|$ and $d_{k}$ represent gain and delay in the forward path, respectively. This delay must be at least one sample, i.e., $d_{k}⩾1$. By substituting (1) into (2) we obtain the transfer function of a closed-loop system from the incoming signal to the loudspeaker signal as follows:

(3) $$S\left(q\right)\;=\;\frac{K\left(q\right)}{1-K\left(q\right)F\left(q\right)}.$$

With the presence of the forward path, the acoustic feedback signal is amplified and looped back into the loudspeaker again and again, which may render the system into unstable conditions. Acoustic feedback is the main problem in HA since it significantly degrades the achievable maximum amplification and output signal quality. The stability of an LTI closed-loop system is based on the Nyquist stability criterion which is stated that a closed-loop system is unstable if conditions for loop gain and loop phase in (4) are met simultaneously.

(4) $$\left\{\begin{array}{c} \begin{array}{cc} \left|K\left(e^{j\omega }\right)F\left(e^{j\omega }\right)\right|\;\geq \;1\\ ∠K\left(e^{j\omega }\right)F\left(e^{j\omega }\right)\;=\;2\pi n, & n\in Z \end{array}, \end{array}\right.$$

where $K\left(e^{j\omega }\right)$ and $F\left(e^{j\omega }\right)$ are the frequency responses of the forward path and the acoustic feedback path, respectively, and $\omega \in \left[0,2\pi \right]$ is the angular frequency. The Nyquist stability criterion in (4) is essential for acoustic feedback control as acoustic feedback control methods effectively try to avoid either one or both of these conditions to be met [3]. Note that an unstable system will result in an unbounded output but due to a natural limiting circuit in the amplifier and loudspeaker it may result in howling.

## 3. Standard AFC Approach

For the sake of simplicity, we assume that incoming signals are stationary and that all AFC systems are discrete and linear time-invariant (LTI). Figure 1 depicts a block diagram of a standard adaptive feedback cancellation system for hearing aids using a single-microphone single-loudspeaker (SMSL). The main idea is to adopt an FIR adaptive filter ($\hat{F}\left(q\right)$) to estimate the true feedback path ($F\left(q\right)$), then the estimated feedback path is utilised to compute the feedback signal estimate, $\hat{v}\left(k\right)$. The error signal, $e\left(k\right)$, is computed by subtracting $\hat{v}\left(k\right)$ from the microphone signal, $m\left(k\right)$. This error signal goes through the forward path ($K\left(q\right)$) where it is delayed and amplified before it feeds into the HA’s loudspeaker. The error signal utilised for an adaptive estimate of the feedback path is computed as follows:

(5) $$e\left(k\right)\;=\;m\left(k\right)\;-\;\hat{v}\left(k\right),$$

where $\hat{v}\left(k\right)\;=\;{\hat{f}}^{T}u\left(k\right)$ is a $L_{\hat{f}}$-dimensional vector, and the vector $\hat{f}\;=\;{\left[{\hat{f}}_{0},{\hat{f}}_{1},\ldots ,{\hat{f}}_{L_{\hat{f}}-1}\right]}^{T}$ denotes the estimated feedback path of length $L_{\hat{f}}$. The $L_{\hat{f}}$-dimensional vector $u\left(k\right)$ is defined as $u\left(k\right)\;=\;{\left[u\left(k\right),u\left(k-1\right),\ldots ,u\left(k-L_{\hat{f}}+1\right)\right]}^{T}$. In HAs, the loudspeaker signal $u\left(k\right)$ is formed by processing the error signal $e\left(k\right)$ using the forward path $K\left(q\right)$, i.e.,

(6) $$u\left(k\right)\;=\;K\left(q\right)e\left(k\right).$$

The transfer function of the closed-loop AFC system is defined as

(7) $$\bar{S}\left(q\right)\;=\;\frac{K\left(q\right)}{1-K\left(q\right)\left[F\left(q\right)-\hat{F}\left(q\right)\right]}.$$

The closed-loop system $\bar{S}\left(q\right)$ is stable if the condition $\left|K\left(e^{j\omega }\right)\left[F\left(e^{j\omega }\right)-\hat{F}\left(e^{j\omega }\right)\right]\right|<1$ is fulfilled. $\hat{F}\left(e^{j\omega }\right)$ is the frequency response of the estimated feedback path.

By minimising the cost function $J\left(\hat{f}\right)\;=\;E\left\{e^{2}\left(k\right)\right\}$ with respect to $\hat{f}$, we obtain an optimal solution as

(8) $${\hat{f}}_{0}\;=\;E{\left\{u\left(k\right)u^{T}\left(k\right)\right\}}^{-1}E\left\{u\left(k\right)m\left(k\right)\right\}.$$

Substituting (1) into (8) yields [1]

(9) $${\hat{f}}_{0}\;=\;f+\underset{bias}{\underset{︸}{R_{u}^{-1}r_{ux}}}.$$

It can be observed that there is a bias in the estimate of feedback path in (9) due to the correlation between the loudspeaker and incoming signals. Thus, the incoming signal acts as a disturbance to the feedback canceller [2]. We recursively approximate the vector ${\hat{f}}_{0}$ using the NLMS algorithm as follows:

(10) $$\hat{f}\left(k\right)\;=\;\hat{f}\left(k-1\right)+\frac{\mu }{u^{T}\left(k\right)u\left(k\right)+\delta_{NLMS}}u\left(k\right)e\left(k\right),$$

where μ is a fixed step-size and $\delta_{NLMS}$ is a small positive value added to avoid division by zero.

In the ideal case where the acoustic feedback path is perfectly estimated, i.e., $\hat{F}\left(q\right)\;=\;F\left(q\right)$, we obtain the loudspeaker signal as a delayed and amplified version of the incoming signal.

## 4. PEM-AFC

**Figure 3:**
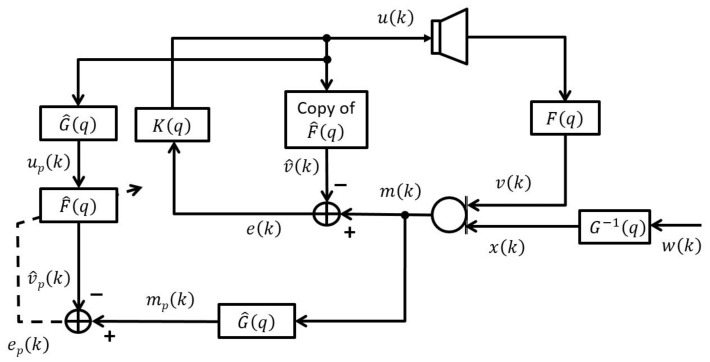
PEM-AFC model.

To address the bias in the feedback path estimate, prediction error method based adaptive feedback cancellation (PEM-AFC) has been proposed in [14] and widely used in both time-domain and frequency-domain AFC approaches. Figure 3 depicts the PEM-AFC model for a SMSL hearing aid [2,14,20]. In the PEM-AFC, first pre-filters are employed to pre-whiten the inputs of the adaptive filter, then the adaptive filter coefficients are recursively updated using pre-whitened signals. In this method, the incoming signal is assumed to be modelled by an autoregressive (AR) process, i.e.,

(11) $$x\left(k\right)\;=\;G^{-1}\left(q\right)w\left(k\right),$$

where $w\left(k\right)$ denotes white Gaussian noise and $G^{-1}\left(q\right)$ is a monic and inversely stable all-pole filter. The loudspeaker and microphone signals are pre-whitened by using $\hat{G}\left(q\right)$. The $\hat{G}\left(q\right)$ is the estimated version of $G\left(q\right)$.

(12) $$m_{p}\left(k\right)\;=\;\hat{G}\left(q\right)m\left(k\right),$$

(13) $$u_{p}\left(k\right)\;=\;\hat{G}\left(q\right)u\left(k\right),$$

(14) $$x_{p}\left(k\right)\;=\;\hat{G}\left(q\right)x\left(k\right),$$

where $m_{p}\left(k\right)$, $u_{p}\left(k\right)$, and $x_{p}\left(k\right)$ represent pre-whitened microphone, pre-whitened loudspeaker, and pre-whitened incoming signals, respectively.

The prediction error signal $e_{p}\left(k\right)$ is defined as

(15) $$e_{p}\left(k\right)\;=\;m_{p}\left(k\right)\;-\;{\hat{f}}^{T}u_{p}\left(k\right),$$

where $u_{p}\left(k\right)\;=\;{\left[u_{p}\left(k\right),u_{p}\left(k-1\right),\ldots ,u_{p}\left(k-L_{\hat{f}}+1\right)\right]}^{T}$ is a $L_{\hat{f}}$-dimensional vector. The IR of the true feedback path in the PEM-AFC can be estimated by minimising the mean square prediction error, $E\left\{e_{p}^{2}\left(k\right)\right\}$,

(16) $$\begin{array}{cc} \min_{\hat{f}}\;E\left\{e_{p}^{2}\left(k\right)\right\} & =\;\min_{\hat{f}}\;E\left\{{\left|m_{p}\left(k\right)-{\hat{f}}^{T}u_{p}\left(k\right)\right|}^{2}\right\}. \end{array}$$

The optimal solution for (16) can be written as follows:

(17) $${\hat{f}}_{0}\;=\;E{\left\{u_{p}\left(k\right)u_{p}^{T}\left(k\right)\right\}}^{-1}E\left\{u_{p}\left(k\right)m_{p}\left(k\right)\right\},$$

(18) $${\hat{f}}_{0}\;=\;f+\underset{bias}{\underset{︸}{R_{u_{p}}^{-1}r_{u_{p}x_{p}}}}.$$

By substituting (11) and (14) into (18), an unbiased estimate of the feedback path can be obtained if the assumption (11) is satisfied, $\hat{G}\left(q\right)\;=\;G\left(q\right)$, and at least one delay in the forward path is available. Furthermore, both the feedback path $F\left(q\right)$ and the AR model $\hat{G}\left(q\right)$ can be identified in closed-loop without adding a probe signal or nonlinearities if the delay in the forward path is not smaller than the length of AR model $\hat{G}\left(q\right)$ [2,14].

The optimal coefficients ${\hat{f}}_{0}$ of the PEM-AFC can be recursively approximated using the NLMS algorithm as

(19) $$\hat{f}\left(k\right)\;=\;\hat{f}\left(k-1\right)\;+\;\frac{\mu }{u_{p}^{T}\left(k\right)u_{p}\left(k\right)+\delta_{NLMS}}u_{p}\left(k\right)e_{p}\left(k\right).$$

## 5. Proposed Method

**Figure 4:**
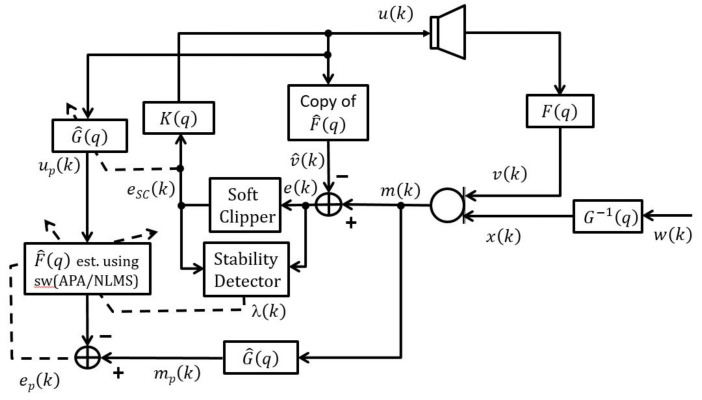
The model of the proposed method.

In this subsection, the structure of the switched prediction error method with soft clipping (swPEMSC) is proposed for adaptive feedback cancellation in hearing aids. Figure 4 illustrates the swPEMSC model. This model is similar to the model of the hybrid NLMS adaptive feedback cancellation algorithm (H-NLMS) using a soft-clipping-based stability detector (SCSD) [44]. The main difference is in the way to update the adaptive filter $\hat{F}\left(q\right)$. In [44], a SCSD is adopted to control the update of the adaptive filter in such a way that the NLMS algorithm is selected when the system is or close to unstable conditions, and the PEMSC-NLMS algorithm is selected when it has converged. This is based on the idea that the NLMS algorithm provides a quick recovery from howling and the PEMSC-NLMS algorithm enables lower misalignment leading to a closer estimate of the true channel. When the feedback is strong, $\hat{v}\left(k\right)$ and $v\left(k\right)$ are not close, and thus the feedback contribution will dominate over the incoming signal, i.e., $\left|v\left(k\right)\;-\;\hat{v}\left(k\right)\right|\;\gg \;\left|x\left(k\right)\right|.$ Inspired by bounded loudspeaker signal u(k), in practice, howling will be produced in this case. By choosing suitable parameters, the H-NLMS can significantly shorten the howling periods and improve the signal quality [44].

**Figure 5:**
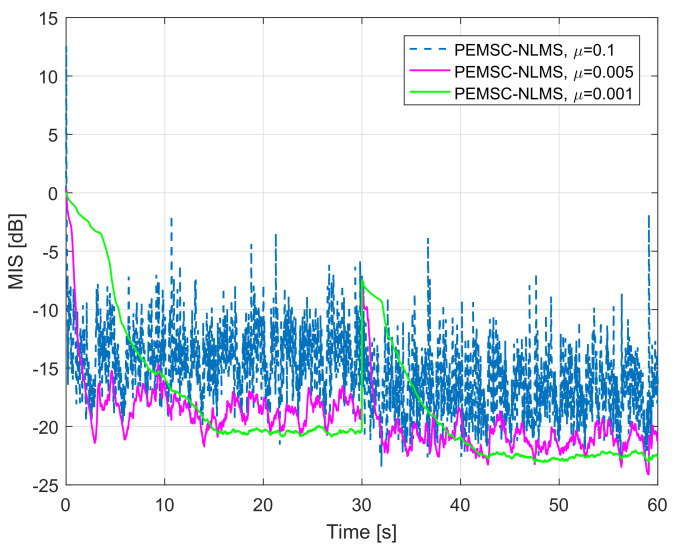
Normalised misalignment of the PEMSC-NLMS with $\mu \in \left[0.1,0.005,0.001\right]$, speech input, feedback path changes from free-field ($F_{1}$) to telephone-near ($F_{2}$).

**Figure 6:**
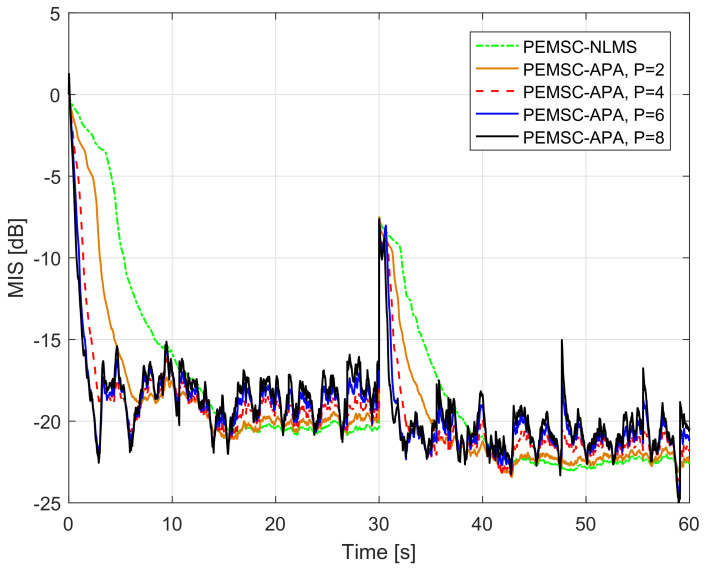
Normalised misalignment of the PEMSC-NLMS, PEMSC-APA with $P\in \left[2,4,6,8\right]$, speech input, feedback path changes from $F_{1}$ to $F_{2}$.

To further improve the convergence/tracking rates as well as steady-state error, while retaining good output sound quality, we propose a new rule to update the feedback path estimate. We observe that standard adaptive algorithms such as LMS, NLMS, and APA suffer from a trade-off between fast convergence/tracking rates and low steady-state error. Particularly, such algorithms yield fast convergence/tracking rates, but high steady-state error when their step-size is large, and vice versa. Figure 5 illustrates the trade-off for AFC using PEMSC-NLMS with different step-size values. In addition, the affine projection algorithm (APA) also exposes to this trade-off with respect to (w.r.t) its projection order (*P*), i.e., the APA provides a fast convergence/tracking, but high steady-state error when *P* is large and vice versa [45,46]. Figure 6 shows that the PEMSC-APA obtains a higher convergence/tracking rate than the PEMSC-NLMS. Moreover, with a fixed step-size the PEMSC-APA converges faster but yields a higher steady-state error when the projection order increases to a certain level, for example, from P=2 to P=6 in the experiment. With P=8, there is almost no improvement in the convergence of PEMSC-APA, but the worse steady-state error is obtained compared to the same experiment with P=6. Therefore, we select P=6 for PEMSC-APA in Experiments 2 and 3.

The proposed method, swPEMSC, is developed to address the above trade-off problems. It is inspired by the SCSD in [44]. In contrast to the authors of [44], we propose to employ SCSD to control a switch between the prediction error method using soft clipping and NLMS (PEMSC-NLMS) and the PEMSC using APA (PEMSC-APA). Specifically, we utilise pre-filters to pre-whiten the inputs of the adaptive filter $\hat{F}\left(q\right)$ leading to lower bias in the estimate of filter coefficients. Then, we apply SCSD to design a switch that allows the adaptive filter to pick up the PEMSC-NLMS algorithm with a small step-size when the system has converged and the PEMSC-APA with a large step-size and projection order when the system is or close to unstable status. As a result, the proposed swPEMSC can achieve a low steady-state misalignment during stable periods and fast convergence/tracking rates to quickly recover from howling during unstable periods. It also solves the mentioned compromise of the APA w.r.t. its projection order, i.e., when *P* increases to a certain level, the swPEMSC achieves faster convergence or tracking rate, while still retaining a low steady-state error.

Simulation results show that the proposed swPEMSC outperforms either PEMSC-NLMS or PEMSC-APA for adaptive feedback cancellation in HAs in terms of normalised misalignment (MIS), added stable gain (ASG) and perceptual evaluation of speech quality (PESQ). It also further improves convergence/tracking ability compared to the H-NLMS [44] with a price of higher computational complexity. Furthermore, the proposed method yields a comparable or better output signal quality (PESQ) compared to all mentioned baselines. In the following, formulations of the swPEMSC will be described in detail. Definitions of the microphone, feedback and error signals are similar to those in (1) and (5), i.e.,

(20) $$m\left(k\right)\;=\;x\left(k\right)\;+\;v\left(k\right),$$

(21) $$\begin{array}{cc} e\left(k\right) & =m\left(k\right)\;-\;\hat{v}\left(k\right)\\ \; & =x\left(k\right)\;+\;\left[v\left(k\right)\;-\;\hat{v}\left(k\right)\right]. \end{array}$$

The pre-whitened microphone, loudspeaker, and error signals are also defined similar to those in (12), (13), and (15), i.e.,

(22) $$m_{p}\left(k\right)\;=\;\hat{G}\left(q\right)m\left(k\right),$$

(23) $$u_{p}\left(k\right)\;=\;\hat{G}\left(q\right)u\left(k\right),$$

(24) $$e_{p}\left(k\right)\;=\;m_{p}\left(k\right)\;-\;{\hat{f}}^{T}u_{p}\left(k\right),$$

where $u_{p}\left(k\right)\;=\;{\left[u_{p}\left(k\right),u_{p}\left(k-1\right),\ldots ,u_{p}\left(k-L_{\hat{f}}+1\right)\right]}^{T}$.

In the proposed method, a soft-clipping (SC) is applied to the error signal yielding the soft-clipping error signal,

(25) $$e_{SC}\left(k\right)\;=\;\alpha \tanh\left(\frac{e\left(k\right)}{\alpha }\right),$$

where α is a scaling parameter. We select α such that the most likely range of the incoming signal lies in the linear range of the tanh-function, i.e., $x\left(k\right)\approx \alpha \tanh\left(\frac{x\left(k\right)}{\alpha }\right)$, thus $e\left(k\right)\;-\;e_{SC}\left(k\right)$ may be utilised to detect instability of the AFC. This SC allows for a controlled nonlinearity on the error signal. In this way, the nonlinearity is known and the AFC can be kept linear. As a result, the feedback cancellation performance is improved. We adopt a SCSD to produce a control signal, $\lambda \left(k\right)$, as

(26) $$\lambda \left(k\right)\;=\;\Gamma \left\{\left|e_{SC}\left(k\right)\;-\;e\left(k\right)\right|\;<\;\gamma \right\},$$

where γ is a decision threshold determining the sensitivity of the detector and Γ is a binary function returning 1 if the inequality holds and 0 if not. The proposed AFC method integrates a SCSD into the update rule of the adaptive filter $\hat{F}\left(q\right)$ such that the PEMSC-NLMS is selected when the system is converged, and the PEMSC-APA is selected when the system is unstable or close to unstable. We set a small step-size value for the PEMSC-NLMS, and a large step-size value for PEMSC-APA aiming at taking advantage of a low steady-state error of the PEMSC-NLMS with a small step-size and fast convergence/tracking rates of the PEMSC-APA with a large step-size. The proposed update rule is defined as follows:

(27) $$\begin{array}{cc} \hat{f}\left(k\right) & =\hat{f}\left(k-1\right)\;+\;\frac{\mu_{1}\lambda \left(k\right)u_{p}\left(k\right)e_{p}\left(k\right)}{u_{p}^{T}\left(k\right)u_{p}\left(k\right)\;+\;\delta_{NLMS}}\\ \; & +\;\mu_{2}\left[1-\lambda \left(k\right)\right]U_{p}\left(k\right){\left[U_{p}^{T}\left(k\right)U_{p}\left(k\right)\;+\;\delta_{APA}I_{P}\right]}^{-1}e_{p}\left(k\right), \end{array}$$

where $I_{P}$ denotes a PxP identity matrix, $\mu_{1}$ and $\mu_{2}$ are fixed step-sizes ($\mu_{2}\gg \mu_{1}$), $\delta_{NLMS}$ and $\delta_{APA}$ denote regularisation parameters of the NLMS and APA, respectively, and $U_{p}\left(k\right)$ is a $L_{\hat{f}}xP$ matrix representing *P* recent loudspeaker signal vectors of length $L_{\hat{f}}$ after being pre-whitened by the pre-filter $\hat{G}\left(q\right)$,

$$U_{p}\left(k\right)\;=\;\left[u_{p,0}\left(k\right)\;u_{p,1}\left(k\right)\;\ldots \;u_{p,P-1}\left(k\right)\right].$$

The loudspeaker signal can be computed as

(28) $$u\left(k\right)\;=\;K\left(q\right)e_{SC}\left(k\right).$$

Besides, the soft-clipping error signal is used to estimate the pre-filter coefficients via Levinson–Durbin algorithm.

## 6. Computational Complexity

In this section, we compare the computational complexity of four considered AFC approaches. The computational complexity for estimating the linear predictor coefficients (LPC) using the autocorrelation matrix and the Levinson–Durbin algorithm is $\frac{5N^{2}+2LN+N}{2L}$ multiplications, where *N* is the AR-model order and *L* is the frame length. Additionally, each pre-whitened signal is computed using *N* multiplications and soft-clipping needs 2 multiplications. Thus the PEMSC requires $M=\frac{5N^{2}+2LN+N}{2L}+2N+2$ multiplications per output sample. For estimating the adaptive filter coefficients using NLMS and APA we need $3L_{\hat{f}}+2$ and $\left(P^{2}+2P\right)L_{\hat{f}}+P^{3}+P$ multiplications, respectively [47], where $L_{\hat{f}}$ denotes the adaptive filter order and *P* is the projection order.

Table 1 summarises the number of real multiplications per output sample [48] for each AFC approach, where we assume that a real multiplication and a real division have equal complexity. It can be seen that the PEMSC-NLMS has the lowest complexity. The computational complexity of the H-NLMS is slightly higher than that of the PEMSC-NLMS. The AFC approaches using the APA like PEMSC-APA and swPEMSC yield higher computational complexity than the approaches using only the NLMS due to a large value of *P*. However, the proposed swPEMSC achieves significant improvement on convergence/tracking rates as well as steady-state error compared to other mentioned AFC approaches. It also provides much higher perceptual speech quality (PESQ score) than the PEMSC-NLMS and PEM-APA, and a comparable PESQ score compared to the H-NLMS for both feedback paths.

## 7. Experimental Results

**Figure 7:**
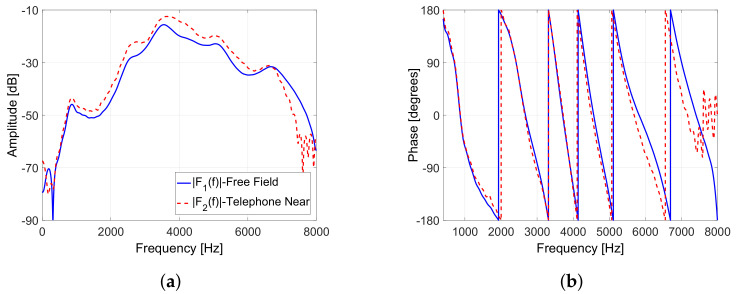
Measured feedback paths: (**a**) Amplitude responses and (**b**) phase.

We use measured feedback paths, so-called free-field ($F_{1}$) and telephone-near ($F_{2}$), corresponding to the case without obstacle between loudspeaker and microphone and the case a telephone placed very close to the ear, respectively [49] for Experiments 1–3. Figure 7 depicts the amplitude and phase responses of these measured feedback paths. It can be observed that the $F_{2}$ feedback path has a higher amplitude response than the $F_{1}$ feedback path due to the effect of the obstacle.

To evaluate the performance of AFC approaches, we use two common metrics: normalised misalignment (MIS) and added stable gain (ASG). The normalised misalignment [20] and added stable gain [20,50] are defined in (29) and (30), respectively.

(29) $${\mathrm{MIS}}_{i}\;=\;10\log{}_{10}\left(\frac{\int {}_{0}^{\pi }{\left|F_{i}\left(e^{j\omega }\right)-e^{-j\omega d_{fb}}{\hat{F}}_{i}\left(e^{j\omega }\right)\right|}^{2}d\omega }{\int {}_{0}^{\pi }{\left|F_{i}\left(e^{j\omega }\right)\right|}^{2}d\omega }\right),$$

(30) $$\begin{array}{ccc} {\mathrm{ASG}}_{i} & = & 10\log{}_{10}\left(\min_{\omega }\frac{1}{{\left|F_{i}\left(e^{j\omega }\right)-e^{-j\omega d_{fb}}\hat{F_{i}}\left(e^{j\omega }\right)\right|}^{2}}\right)\\ & & -10\log{}_{10}\left(\min_{\omega }\frac{1}{{\left|F_{i}\left(e^{j\omega }\right)\right|}^{2}}\right), \end{array}$$

where *i* is the feedback path index (i=1,2), $F_{i}\left(e^{j\omega }\right)$ and ${\hat{F}}_{i}\left(e^{j\omega }\right)$ denote frequency responses of the *i*th true and the *i*th estimated feedback paths at the normalised angular frequency ω respectively and $d_{fb}$ denotes a delay in the feedback canceller’s path. The lower value of MIS and higher value of ASG indicate the better AFC approach.

In addition, perceptual evaluation of speech quality (PESQ) [51] is utilised to evaluate the quality of the speech signal. The PESQ ranges from −0.5 to 4.5, where the value of −0.5 indicates poor speech quality and the value of 4.5 indicates the highest speech quality. For the PESQ measures, the incoming signal $x\left(k\right)$ and the loudspeaker signal $u\left(k\right)$ are chosen as the reference and test signals, respectively. We evaluate the convergence/tracking rates of AFC methods based on the necessary time (namely, $\tau {}_{\kappa_{i}}$) for each method to reach a certain level of misalignment (namely $\kappa_{i}$) corresponding to the feedback path $F_{i}$.

The following parameters are selected for all simulations: forward path delay $d_{k}=96$ samples, delay of the feedback canceller’s path $d_{fb}=1$ sample, and regularisation parameters $\delta_{NLMS}\;=\;\delta_{APA}\;=\;10^{-6}$. Lengths of the true and estimated feedback paths are $L_{f}=100$ and $L_{\hat{f}}=64$, respectively. For the pre-filter estimate, $\hat{G}\left(q\right)$, a 20-order AR model of the incoming signal is computed for every frame of 160 samples by using the Levinson–Durbin algorithm [52]. For experiments 1–3, the forward path gain $\left|K\right|=30$ dB and sampling frequency $f_{s}=16$ kHz are chosen.

**Figure 8:**
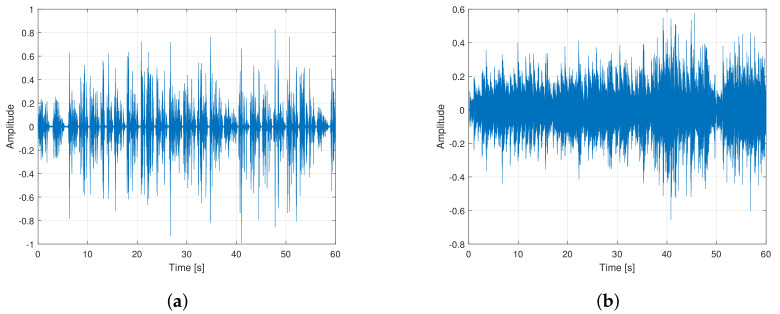
Incoming signals: (**a**) Concatenated speech and (**b**) music.

We compare the proposed AFC approach to state-of-the-art baselines such as PEMSC-NLMS, PEMSC-APA (with different projection order), and H-NLMS [44] using different types of incoming signals, e.g., speech and music signals. Figure 8 shows the recorded speech and music signals used as incoming signals in experiments 1–3. The length of these signals is truncated to 60 s. To evaluate the tracking ability of AFC approaches, a sudden change of the feedback path from ($F_{1}$) to ($F_{2}$) is employed after half of the simulation time. The following step-sizes are selected for AFC approaches to ensure that each AFC approach achieves its best performance.

- For PEMSC-NLMS: μ=0.001 for Experiments 1–3 and μ=0.002 for Experiment 4.
- For PEMSC-APA: μ=0.0008.
- For H-NLMS and swPEMSC: $\mu_{1}=0.0008$, $\mu_{2}=0.8$.

**Figure 9:**
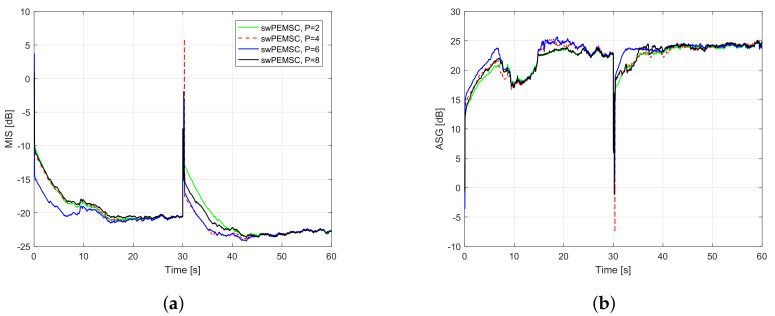
Performance of the proposed method with $P\in \left[2,4,6,8\right]$, speech input, feedback path changes from free-field ($F_{1}$) to telephone-near ($F_{2}$): (**a**) MIS; (**b**) ASG.

**Figure 10:**
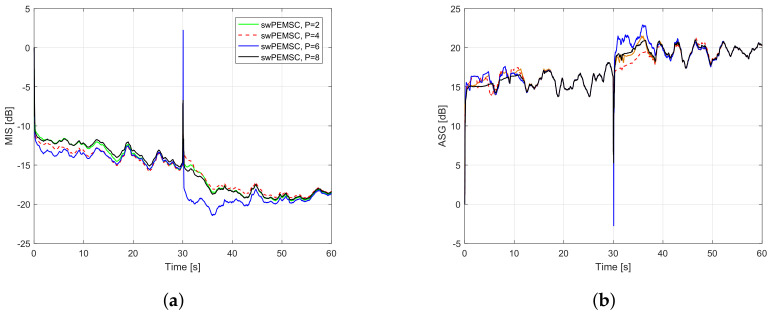
Performance of the proposed method with $P\in \left[2,4,6,8\right]$, music input, feedback path changes from free-field ($F_{1}$) to telephone-near ($F_{2}$): (**a**) MIS; (**b**) ASG.

*Experiment 1*: This experiment aims at finding a suitable value of projection order such that the proposed method achieves a good performance as well as an acceptable complexity. Figure 9 demonstrates the performance of the proposed approach with different projection order $P\in \left[2,4,6,8\right]$ in term of MIS and ASG. It can be seen that the convergence rate of the swPEMSC is quite similar for P=2,4,8. The tracking rate is improved with an increase of *P* from 2 to 4 or 6. Further increasing *P* degrades the system performance. For example, the tracking rate with P=8 is lowered than that with P=4 or 6. In the experiment, we found that P=6 is the best choice as it allows the swPEMSC to achieve the highest convergence/tracking rate while maintaining a similar steady-state misalignment. Similar observations are also reported when using music as the incoming signal, cf. Figure 10. Therefore, we select P=6 for the proposed swPEMSC.

*Experiment 2:* This experiment aims at evaluating the performance of the proposed swPEMSC for recorded speech [30] as the incoming signal. The feedback paths depicted in Figure 7 are selected. We suddenly change the feedback path from $F_{1}$ to $F_{2}$ after 30 s. The performance of the proposed swPEMSC is evaluated in terms of normalised misalignment (MIS), added stable gain (ASG), signal quality (PESQ) and the necessary time ($\tau {}_{\kappa_{i}}$).

**Figure 11:**
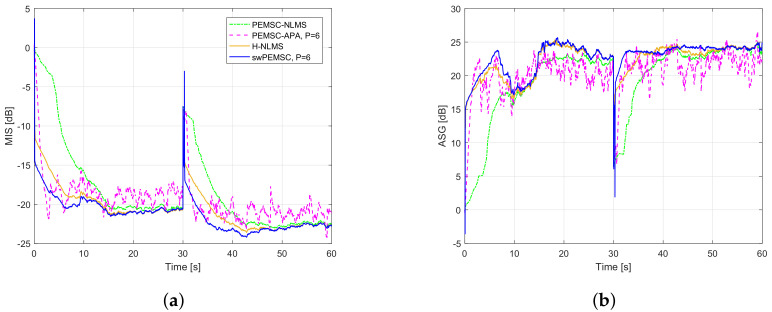
Compare performance of the proposed method with state-of-the art AFC methods using speech input, a sudden change of the feedback path from free-field ($F_{1}$) to telephone-near ($F_{2}$) after 30 s: (**a**) MIS; (**b**) ASG.

Figure 11 compares the performance of the proposed swPEMSC with state-of-the-art baselines such as PEMSC-NLMS, PEMSC-APA (with P=6) and H-NLMS. It can be seen that the PEMSC-APA converges quicker than the PEMSC-NLMS. It also tracks a sudden change of the feedback path from free-field ($F_{1}$) to telephone-near ($F_{2}$) quicker than the PEMSC-NLMS. However, a higher steady-state error with larger variations can be observed from MIS and ASG of the PEMSC-APA. The H-NLMS yields faster initial convergence and tracking rates than the PEMSC-APA while providing a lower steady-state error compared to both PEMSC-NLMS and PEMSC-APA. The proposed method outperforms all mentioned AFC methods. Particularly, it achieves the fastest convergence/tracking rates and the lowest steady-state error. It also provides the highest ASG, especially during the periods in which the system has converged, for example, the periods between 15 s and 30 s corresponding to the feedback path $F_{1}$ and between 45 s and 60 s corresponding to the feedback path $F_{2}$.

Table 2 summarises the comparison of the swPEMSC with baselines in terms of the necessary time in seconds ($\tau {}_{\kappa_{i}}$) for each AFC approach to reach $\kappa_{i}$ dB level of misalignment, average normalised misalignment in dB, and average added stable gain in dB corresponding to the *i*th feedback path (i.e., $\tau {}_{\kappa_{i}}$, ${\bar{MIS}}_{i}$, ${\bar{ASG}}_{i}$), respectively. For recorded speech incoming signal [30], we choose $\kappa_{1}=-15$ dB, $\kappa_{2}=-16$ dB. The best values are indicated in bold. It can be seen that the PEMSC-APA has higher ${\bar{MIS}}_{i}$, ${\bar{ASG}}_{i}$ and much smaller $\tau {}_{\kappa_{i}}$ (i=1,2) than those of the PEMSC-NLMS. The H-NLMS obtains further improvement in ${\bar{MIS}}_{i}$, ${\bar{ASG}}_{i}$, while retaining similar $\tau {}_{\kappa_{2}}$ and a bit higher $\tau {}_{\kappa_{1}}$ compared to the PEMSC-APA. Among all mentioned AFC approaches, the proposed swPEMSC achieves the best values for all metrics. Specifically, the proposed approach yields approximately 4 dB gain on ${\bar{MIS}}_{1}$ and ${\bar{ASG}}_{1}$, 2.5 dB gain on ${\bar{MIS}}_{2}$ and ${\bar{ASG}}_{2}$ compared to the PEMSC-NLMS. It also yields approximately 2 dB gain on ${\bar{MIS}}_{i}$ and ${\bar{ASG}}_{i}$ for both feedback paths compared to the PEMSC-APA, and 0.7 dB improvement in ${\bar{MIS}}_{i}$ and 0.5 dB improvement in ${\bar{ASG}}_{i}$ compared to the H-NLMS. Moreover, the proposed approach needs only 0.3 s to reach −15 dB of misalignment (for $F_{1}$) while the PEMSC-NLMS, PEMSC-APA, and H-NLMS need around 8.3 s, 2.5 s, and 1.7 s, respectively. Similar observations are reported for the case using $F_{2}$. Note that a small value of $\tau {}_{\kappa_{1}}$ implies a faster convergence rate and a small value of $\tau {}_{\kappa_{2}}$ means a faster tracking rate.

We evaluate the speech quality of the compared AFC approaches using the PESQ measure (cf. Table 3). This table shows that H-NLMS and swPEMSC outperform PEMSC-NLMS and PEMSC-APA in terms of PESQ. The swPEMSC yields a slightly higher PESQ for the free-field feedback path ($F_{1}$) but gets a small drop in PESQ for the telephone-near feedback path ($F_{2}$) compared to the H-NLMS. Both H-NLMS and swPEMSC obtain high perceptual speech quality with PESQ scores from 3.7 to 4.2. We observe that the misalignment of swPEMSC yields a higher peak (a sign of howling) than that of the H-NLMS after a sudden change of feedback path. However, this high peak lasts only for a very short time before the system quickly returns to a stable state (see Figure 11a). That may be the reason for a small reduction in the ${\mathrm{PESQ}}_{2}$ of the swPEMSC compared to that of H-NLMS. To verify this observation, we compute ${\mathrm{PESQ}}_{2}$ with incoming signal from 32 s to 60 s (skipping the first 2 s that may contain howling for the H-NLMS and swPEMSC). In this case, the ${\mathrm{PESQ}}_{2}$ scores for PEM-NLMS, PEM-APA, H-NLMS, and swPEMSC are 3.571, 4.084, 4.224, and 4.291, respectively. This result means that the three last methods achieve good signal quality (PESQ > 4), but the proposed approach obtains the highest PESQ for the period of the signal without the howling effect. This result matches well with Figure 11a, where the three last methods show quick tracking rates. The ${\mathrm{PESQ}}_{2}$ of the PEMSC-NLMS is much lower since this method tracks the change of feedback path slower than other methods resulting in a part of howling still available in the ${\mathrm{PESQ}}_{2}$ computing period (32 s–60 s).

**Figure 12:**
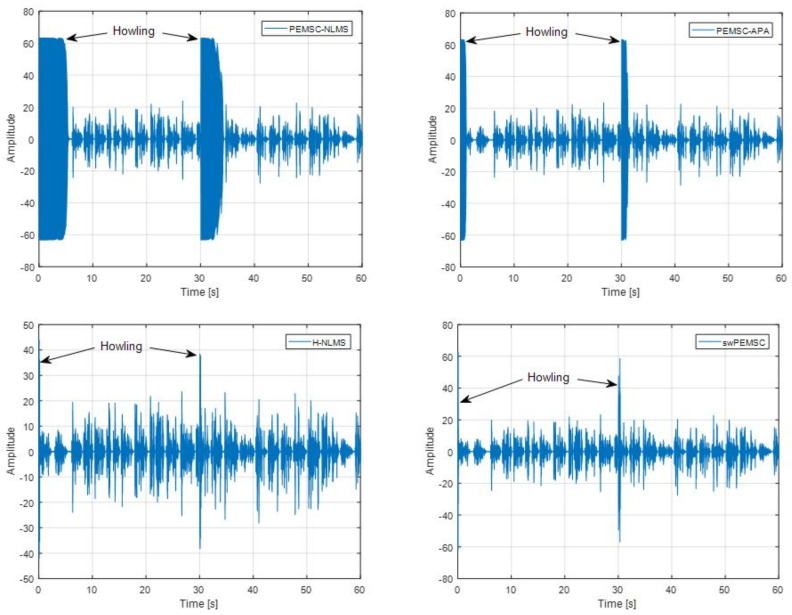
Compare howling periods in output signal of the proposed approach with baselines, speech incoming signal, a sudden change of the feedback path from $F_{1}$ to $F_{2}$ after 30 s.

Those results are consistent with an observation of howling periods in Figure 12. We can see that the PEMSC-APA yields shorter howling than the PEMSC-NLMS. The howling periods of the H-NLMS and the proposed swPEMSC are comparable, but they are much shorter than those of the PEMSC-APA. Therefore, the swPEMSC and H-NLMS can recover from howling periods quicker than the PEMSC-APA and PEMSC-NLMS.

*Experiment 3:* In this experiment, we evaluate the proposed swPEMSC using recorded music [24] as the incoming signal. The recorded music is a segment of the song “Imagine” by John Lennon. We select the feedback paths depicted in Figure 7 and suddenly change the feedback path after half of the simulation time. We set $\kappa_{1}=-11$ dB and $\kappa_{2}=-14.5$ dB.

**Figure 13:**
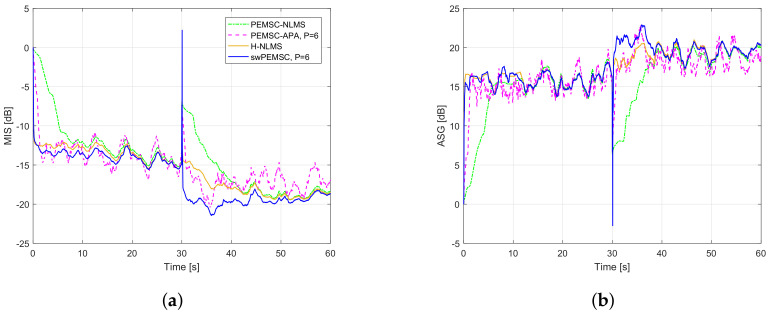
Performance of the proposed method state-of-the art AFC methods using music input, a sudden change of the feedback path from free-field ($F_{1}$) to telephone-near ($F_{2}$) after 30 s: (**a**) MIS; (**b**) ASG.

Figure 13 demonstrates the performance of the proposed approach in comparison with other baselines. The proposed swPEMSC outperforms all considered AFC approaches. In particular, it achieves faster convergence/tracking rates and lower steady-state error than other baselines. Those observations are consistent with the results shown in Table 2 for the music incoming signal. It is shown that the proposed approach obtains the best values for most of metrics such as ${\bar{MIS}}_{1}$,${\bar{MIS}}_{2}$, ${\bar{ASG}}_{2}$, $\tau {}_{\kappa_{1}}$, and $\tau {}_{\kappa_{2}}$. The H-NLMS obtains the best value for ${\bar{ASG}}_{1}$, while the ${\bar{ASG}}_{1}$ of swPEMSC is comparable with that of the H-NLMS.

**Figure 14:**
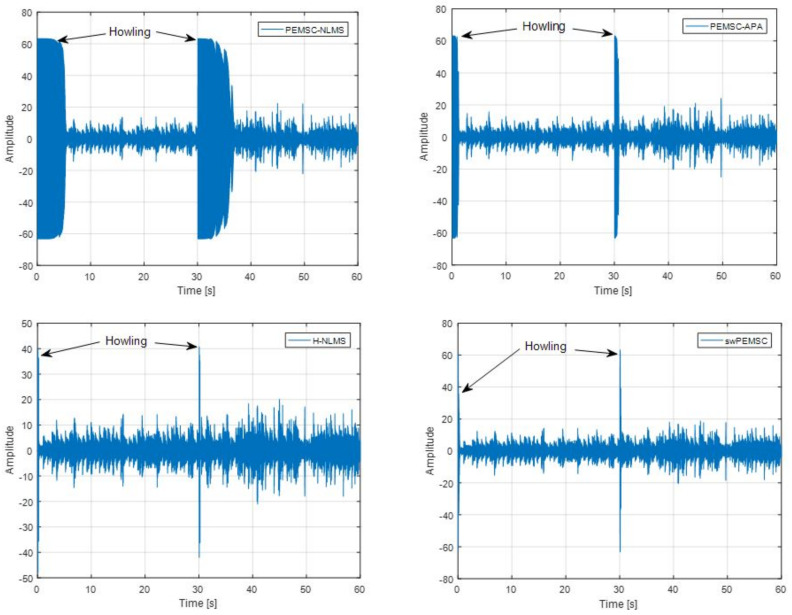
Compare howling periods in output signal of the proposed approach with baselines, music incoming signal, a sudden change of the feedback path from $F_{1}$ to $F_{2}$ after 30 s.

Figure 14 compares howling periods of the proposed approach with those of baselines. It can be seen that the swPEMSC can recover from howling quicker than the PEMSC-NLMS and PEMSC-APA. Its howling length is comparable with that of the H-NLMS.

*Experiment 4:* This experiment is conducted to verify the robustness of the proposed approach against different input signals and feedback paths. In particular, we evaluate the proposed swPEMSC using five segments of concatenated speech as the incoming signals and a new feedback path.

**Figure 15:**
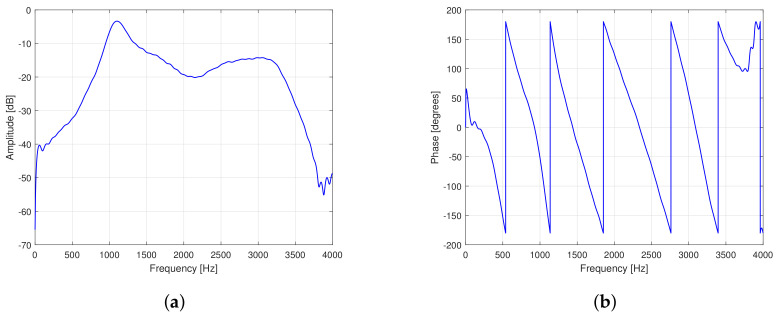
The measured feedback path for Experiment 4: (**a**) Amplitude response and (**b**) phase.

**Figure 16:**
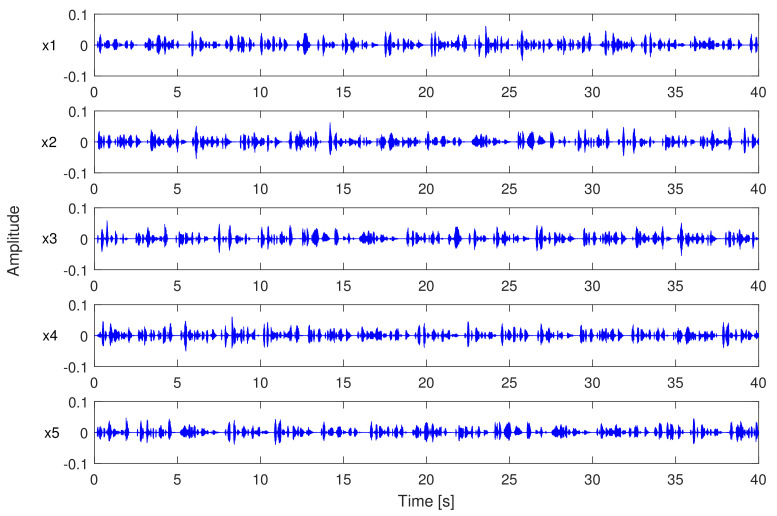
Speech segments mentioned in Experiment 4.

Note that the incoming signals in this experiment are not recorded. Each speech segment is generated by randomly selecting and concatenating speech utterances extracted from NOIZEUS database [51]. The length of each segment is 40 s. The measured feedback path [20] is selected for this experiment. This measured feedback path ($F_{1}$) and the five segments of speech incoming signals are presented in Figure 15 and Figure 16, respectively. The sampling frequency for this experiment is 8 kHz. To evaluate the tracking ability of AFC methods we produce the second feedback path ($F_{2}$) by right shifting $F_{1}$ by 12 samples. In this experiment, we set $\left|K\right|=12$ dB for the forward path gain, $\kappa_{1}=\kappa_{2}=-13.5$ dB, and step-size μ=0.002 for the PEMSC-NLMS. We also select P=2 for the PEMSC-APA P=6 for the swPEMSC as they allow the best performance for the corresponding approach. Other parameters are set the same as those in Experiment 2.

**Figure 17:**
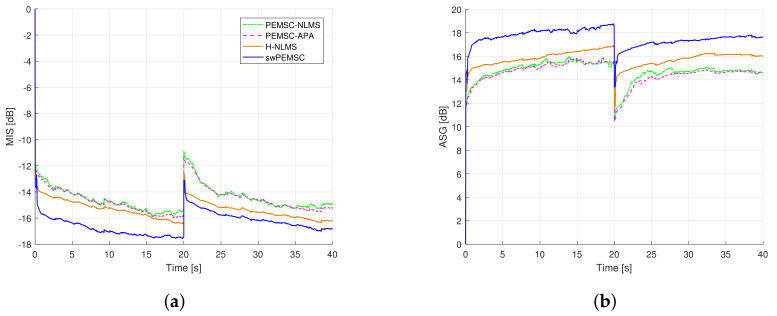
Compare performance of the proposed method with state-of-the art AFC methods using 5 segments of speech input, a sudden change of the feedback path from $F_{1}$ to $F_{2}$ after 20 s, MIS (**a**) and ASG (**b**) were average values computed over 5 segments of speech input.

Figure 17 shows the average MIS and ASG computed over 5 segments of speech input. The feedback path abruptly changes from $F_{1}$ to $F_{2}$ after 20 s. It can be seen that the PEMSC-APA and PEMSC-NLMS obtain similar performance, while the H-NLMS achieves quicker convergence/tracking rates and also lower steady-state error. Higher average ASGs for both feedback paths are also observed for the H-NLMS compared to those of PEMSC-NLMS and PEMSC-APA. As expected the swPEMSC outperform all baselines. Those observations match well with the results in Table 2, where the proposed swPEMSC achieves the highest ${\bar{MIS}}_{i}$ and ${\bar{ASG}}_{i}$. It also obtains comparable $\tau {}_{\kappa_{i}}$ compared to the H-NLMS, but those values much lower compared to the PEMSC-APA and PEMSC-NLMS.

Table 3 shows the average PESQ scores over five segments of speech input, where ${\mathrm{PESQ}}_{1}$ and ${\mathrm{PESQ}}_{2}$ are measured over the last 18 s of speech segments corresponding to the feedback path $F_{1}$ and $F_{2}$, respectively. It is observed that the ${\mathrm{PESQ}}_{1}$ scores of all mentioned AFC methods are very high (approximately 4.1), which reflect the fact that after the first 2 s all AFC methods have converged. These results are consistent with the measures of $\tau {}_{\kappa_{1}}$ in Table 2 which shows that all AFC methods during the period of the feedback path $F_{1}$ need around 2 s or less to reach −13.5 dB of misalignment. When the feedback path suddenly changes from $F_{1}$ to $F_{2}$, the PEMSC-NLMS and PEMSC-APA need approximately 3 s and 2.8 s to reach that level of misalignment, respectively. It may be the reason for a reduction in ${\mathrm{PESQ}}_{2}$ of those methods since the system may still partly unstable during the period ${\mathrm{PESQ}}_{2}$ computed. In contrast, the H-NLMS and swPEMSC require a very short time to reach that level of misalignment for both feedback paths, resulting in very high ${\mathrm{PESQ}}_{2}$ scores (approximately 4.1).

Although the proposed swPEMSC has comparable signal quality compared to the H-NLMS, it achieves the lowest average MIS, the highest ASG, and faster convergence/tracking rates for most scenarios.

Note that in experiments 2 and 3 the average misalignment (${\bar{MIS}}_{i}$) and average added stable gain (${\bar{ASG}}_{i}$) corresponding to the *i*th feedback path (i=1,2) are computed over 30 s (i.e., 480,000 samples) of each realisation, while in experiment 4 those values are computed over 20 s (i.e., 160,000 samples).

## 8. Discussion

In this study, the MIS, ASG, and PESQ measures are adopted to evaluate the performance of different AFC approaches, namely, the swPEMSC, H-NLMS, PEMSC-APA, and PEMSC-NLMS for different types of incoming signals and an abrupt change of the feedback path. Moreover, this study evaluates the convergence/tracking rates based on the needed time for each considered approach reaching a certain level of normalised misalignment. Experimental results indicate that the proposed swPEMSC achieves a further improvement in the initial convergence and re-convergence than the state-of-the-art H-NLMS for most scenarios. The reason for faster re-convergence is that the swPEMSC uses an APA adaptive filter update with a high step-size and an optimised project order when unstable/howling status is detected, whereas the H-NLMS uses a standard NLMS. Furthermore, the swPEMSC and H-NLMS outperform the PEMSC-APA and PEMSC-NLMS in terms of convergence/tracking rates as well as average MIS and average ASG.

The results also show that there is a dependency of the projection order (for AFC approaches using the APA algorithm) on the performance. This is expected because the projection order of an APA algorithm depends on the signal characteristics. It is observed that the proposed approach employing a fixed pair of step-sizes $\mu_{1}=0.0008$, $\mu_{2}=0.8$, and a projection order of 6 (P=6) provides better performance than itself with P=2,4,8 for both speech and music input signals as well as for a sudden change of the feedback path.

Generally, the proposed swPEMSC achieves much better speech quality than PEMSC-APA and PEMSC-NLMS in most mentioned scenarios due to its fast convergence and tracking abilities. It also yields similar speech quality compared to the H-NLMS.

## 9. Conclusions

In this paper, we propose a new and practical way to improve the AFC performance in open-fit hearing aids. The proposed swPEMSC is developed based on a new update rule for estimating adaptive filter coefficients. This update rule allows a switch from the PEMSC-NLMS to the PEMSC-APA when the system goes from a converged status to an unstable/howling status, and vice versa. This switch is controlled by a control signal produced by using the SCSD. Experimental results show that the proposed swPEMSC outperforms other state-of-the-art AFC approaches such as the PEMSC-NLMS, PEMSC-APA, and H-NLMS for different types of incoming signals (e.g., speech and music) and an abrupt change of feedback paths. In particular, the proposed approach achieves a significant performance improvement in terms of convergence/tracking rates, average MIS and average ASG compared to baselines in most scenarios. It also obtains high perceptual speech quality. The PESQ score as well as the ability to recover from the instability/howling of the proposed approach are comparable to those of the H-NLMS but much better than those of the PEMSC-NLMS and PEMSC-APA. However, the improvements from using swPEMSC come at an increased cost in computational complexity.

## Figures and Tables

**Table 1:** Computational complexity per output sample.

AFC Methods	Computational Complexity	#
PEMSC-NLMS	$M+3L_{\hat{f}}+2$	263
PEMSC-APA	$M+\left(P^{2}+2P\right)L_{\hat{f}}+P^{3}+P$	3363
H-NLMS	$M+2\left(3L_{\hat{f}}+2\right)$	457
swPEMSC	$M+3L_{\hat{f}}+2+\left(P^{2}+2P\right)L_{\hat{f}}+P^{3}+P$	3557

A numerical value is given for N=20, L=160, $L_{\hat{f}}=64$, and P=6.

**Table 2:** Evaluate performance of PEMSC-NLMS, PEMSC-APA, H-NLMS, and swPEMSC for different types of the incoming signals, feedback path changes from $F_{1}$ to $F_{2}$ after half of the simulation time, $\kappa_{1}=-15$ dB, $\kappa_{2}=-16$ dB (for recorded speech input); $\kappa_{1}=-11$ dB, $\kappa_{2}=-14.5$ dB (for recorded music input); and $\kappa_{1}=\kappa_{2}=-13.5$ dB (for 5 segments of speech input).

AFC Methods	Incoming Signals	${\bar{\mathrm{MIS}}}_{1}$ [dB]	${\bar{\mathrm{ASG}}}_{1}$ [dB]	$\tau {}_{\kappa_{1}}$ [s]	${\bar{\mathrm{MIS}}}_{2}$ [dB]	${\bar{\mathrm{ASG}}}_{2}$ [dB]	$\tau {}_{\kappa_{2}}$ [s]
PEMSC-NLMS		−16.023	17.656	8.312	−20.115	21.194	4.780
PEMSC-APA	recorded speech	−18.176	19.586	1.545	−20.562	21.385	1.180
H-NLMS [44]		−19.278	21.260	2.715	−21.750	23.175	1.186
swPEMSC		**−20.016**	**21.786**	**0.304**	**−22.487**	**23.638**	**0.367**
PEMSC-NLMS		−11.493	14.066	6.540	−16.568	17.354	6.149
PEMSC-APA	recorded music	−13.341	14.970	1.205	−17.073	18.406	0.779
H-NLMS [44]		−13.358	**15.860**	0.193	−17.946	19.278	0.441
swPEMSC		**−13.847**	15.804	**0.144**	**−19.465**	**19.837**	**0.105**
PEMSC-NLMS		−14.655	15.034	2.103	−14.339	14.424	3.088
PEMSC-APA	5 speech segments	−14.768	14.906	1.775	−14.417	14.175	2.795
H-NLMS [44]		−15.254	15.897	**0.203**	−15.446	15.662	0.169
swPEMSC		**−16.810**	**17.936**	0.251	**−16.043**	**17.227**	**0.131**

**Table 3:** PESQ measures of the PEMSC-NLMS, PEMSC-APA, H-NLMS, and swPEMSC with a sudden change of feedback paths from $F_{1}$ to $F_{2}$ after half of the simulation time, recorded speech and 5 segments of speech input as incoming signals.

AFC Methods	Incoming Signals	${\mathrm{PESQ}}_{1}$	${\mathrm{PESQ}}_{2}$
PEM-NLMS		1.652	3.013
PEM-APA	recorded speech	2.067	3.448
H-NLMS [44]		4.167	**4.047**
swPEMSC		**4.218**	3.729
PEM-NLMS		4.132	1.646
PEM-APA	5 speech segments	4.124	1.890
H-NLMS [44]		4.077	**4.129**
swPEMSC		**4.134**	4.075

## Data Availability

The data presented in this study are available in the Supplementary Materials here.

## Supplementary Materials

The following are available online at https://www.mdpi.com/2039-4349/11/3/37/s1.
