# Supplementary material for: A Switched Algorithm for Adaptive Feedback Cancellation Using Pre-Filters in Hearing Aids
Source: Audiol Res. 2021 Aug 9;11(3):389–409. doi: 10.3390/audiolres11030037 (PMC8395445; doi:10.3390/audiolres11030037)
Supplement: Supplementary file 1 [file audiolres-11-00037-s001.zip › Code_for_JAR/Code_for_JAR/CommonFunc/Obj_estimate/readme.pdf]

```
This folder contains implementations of objective measures (Chapters 10 and 11):

        MATLAB file     Description                                     Reference
-----------------------------------------------------------------------------------
        comp_snr.m      Overall and segmental SNR                       [1]

        comp_wss.m      Weighted-spectral slope metric                  [2]

        comp_llr.m      Likelihood-ratio measure                        [3]

        comp_is.m       Itakura-Saito measure                           [3]
        comp_cep.m      Cepstral distance measure                       [4]
        comp_fwseg      Freq. weighted segm. SNR (fwSNRseg)             [5],Chap 11,
                                                                        Eq. 11.5
        comp_fwseg_variant   Frequency-variant fwSNRseg measure         Chapter 10,
                                                                        Eq. 10.24
        comp_fwseg_mars     Frequency variant fwSNRseg measure          Chap 10,
                            based on MARS analysis                      Sec. 10.5.4


        pesq.m          PESQ measure                                    [6]

        composite.m     A composite measure                             [7]


        addnoise_asl.m  Adds noise to the clean signal at specified SNR
                        based on active speech level.                   [8]

---------------------------------------------------------------------------------
USAGE

>>   [snr_mean, segsnr_mean]= compSNR(cleanFile.wav, enhdFile.wav);
      where 'snr_mean' is the global overall SNR and 'segsnr_mean' is the
      segmental SNR.

>>   wss_mean = comp_wss(cleanFile.wav, enhancedFile.wav);

>>   llr_mean= comp_llr(cleanFile.wav, enhancedFile.wav);

>>    is_mean = comp_is(cleanFile.wav, enhancedFile.wav);

>>    cep_mean = comp_cep(cleanFile.wav, enhancedFile.wav);

>>    fwSNRseg = comp_fwseg(cleanFile.wav, enhancedFile.wav);

>>    [SIG,BAK,OVL]=comp_fwseg_variant(cleanFile.wav, enhancedFile.wav);
        where   'SIG' is the predicted rating of speech distortion,
                'BAK' is the predicted rating of background noise distortion,
                'OVL' is the predicted rating of overall quality.

>>    [SIG,BAK,OVL]=comp_fwseg_mars(cleanFile.wav, enhancedFile.wav);

>>    pesq_mean = pesq(cleanFile.wav, enhancedFile.wav);
               Only sampling frequencies of 8000 Hz or 16000 Hz are supported.

>>    [Csig,Cbak,Covl]=composite(cleanFile.wav, enhancedFile.wav);
        where   'Csig' is the predicted rating of speech distortion,
                'Cbak' is the predicted rating of background noise distortion,
                'Covl' is the predicted rating of overall quality.

>>      addnoise_asl(cleanfile.wav, noisefile.wav, outfile.wav, SNRlevel)

---------------------------------------------------------------------------
```

REFERENCES:

[1] Hansen, J. and Pellom, B. (1998). An effective quality evaluation protocol for speech enhancement algorithms. Inter. Conf. on Spoken Language Processing, 7(2819), 2822

[2] Klatt, D. (1982). Prediction of perceived phonetic distance from critical band spectra. Proc. IEEE Int. Conf. Acoust. , Speech, Signal Processing, 7, 1278-1281.

[3] Quackenbush, S., Barnwell, T., and Clements, M. (1988). Objective measures of speech quality. NJ: Prentice-Hall, Eaglewood Cliffs.

[4] Kitawaki, N., Nagabuchi, H., and Itoh, K. (1988). Objective quality evaluation for low bit-rate speech coding systems. IEEE J. Select. Areas in Comm., 6(2), 262-273.

[5] Tribolet, J., Noll, P., McDermott, B., and Crochiere, R. E. (1978). A study of complexity and quality of speech waveform coders. Proc. IEEE Int. Conf. Acoust. , Speech, Signal Processing, 586-590.

[6] ITU (2000). Perceptual evaluation of speech quality (PESQ), and objective method for end-to-end speech quality assessment of narrowband telephone networks and speech codecs. ITU-T Recommendation P. 862

[7] Hu, Y. and Loizou, P. (2006). Evaluation of objective measures for speech enhancement. Proc. Interspeech

[8] ITU-T (1993). Objective measurement of active speech level. ITU-T Recommendation P. 56

Copyright (c) 2006 by Philipos C. Loizou
$Revision: 0.0 $ $Date: 07/30/2006 $
